# How to improve interest for undergraduate nursing students working in primary health care

**DOI:** 10.1017/S1463423623000233

**Published:** 2023-05-12

**Authors:** Beate André, Therese Antonsen, Sølvi Karin Romarheim Akslen, Wenche Bergseth Bogsti

**Affiliations:** 1 Department of Public Health and Nursing, Norwegian University of Science and Technology (NTNU), 7491 Trondheim, Norway; 2 NTNU Center for Health Promotion Research, 7491 Trondheim, Norway; 3 Ålesund Municipality, Health, and Care, Ålesund 6025, Norway; 4 Department of Health Sciences in Gjøvik, Norwegian University of Science and Technology (NTNU), N-2802 Gjøvik, Norway

**Keywords:** clinical placement, nursing education, primary health care, supervision

## Abstract

**Aim:**

To explore the association between the implementation of a new model of supervision and the impact of undergraduate nursing students’ interest in working in primary health care.

**Background:**

There is a need for more nurses in primary health care. To influence undergraduate nursing students to work in primary health care after graduation, the experience of their clinical practice in primary health care must be rewarding. In this study, we have implemented an alternative model of supervision for undergraduate nursing students in clinical practice, called ‘strengthened supervision during clinical practice’. In this model, lectures from the university are responsible for giving support and tutoring the nurse supervisor in primary health care.

**Method:**

Undergraduate nursing students in Norway (69) participated in an implementation of a new model for supervision in clinical practice. Thirty-one completed a questionnaire consisting of 15 questions. The questionnaire was analyzed using descriptive analyses.

**Finding:**

Undergraduate nursing students positively evaluated interactions with their fellow undergraduate nursing students in the primary health care setting. The undergraduate nursing students reported the nurse supervisor as most important for their perception of the practice site, followed by the work environment and their peers. When asked where they planned to work after graduation, very few undergraduate nursing students selected primary health care. It seems like aspects of the new model, ‘strengthened supervision during clinical practice’ are successful, but further research must be undertaken to explore whether this new model continues to be successful.

## Background

Undergraduate nursing students require tutoring on clinical tasks in clinical practice which also provides them with a good learning environment (Warne *et al*., 2010). Earlier findings suggest that factors such as a positive learning environment, collaboration, reassurance, and clinical supervisor support are important aspects for students to learn and train on practical tasks (Warne *et al*., 2010; Hayes *et al*., 2006). For beginner nursing student’ experiences in the clinical learning process is influenced mainly by personnel in clinical learning, but also the peers a student has are crucial for the learning process (Arkan *et al*., 2018; Serçekuş and Başkale, 2016). Research has shown that undergraduate nursing students learn professional roles, skills, knowledge, and behaviors in the practical placement and that it is possible to influence undergraduate nursing students in their first year (Cooper *et al*., 2015; Hanson *et al*., 2020; Chesser-Smyth and Long, 2013).

Earlier findings have indicated that undergraduate nursing students have perceived their nurse supervisors in both a positive and negative way (Noviani *et al*., 2022). In their clinical placement undergraduate nursing students may face some difficulties in their relationship and collaboration with the nurse supervisor (Warne *et al*., 2010). On the other hand, the nurse supervisor may need more expertise and training in supervising undergraduate nursing students (Struksnes *et al*., 2012). There is a need to improve the education and resources available to enhance the role of nurse supervisors when they supervise undergraduate nursing students (Leonardsen *et al*., 2021; Noviani *et al*., 2022). Clinical training intends to improve students’ critical reflection and decision-making abilities and strengthen their self-confidence (Arkan *et al*., 2018). Issues with both students’ and nurse supervisors’ challenges and lack of experience and training in critical reflection may severely influence the students learning and critical reflection (Zlamal *et al*., 2021; Arkan *et al*., 2018).

European collaboration and the Bologna process have influenced changes in nursing education, and there have been strategic changes in nurse education in numerous countries (Lahtinen *et al*., 2014). Undergraduate nursing education has developed into an academic education, including 3-year full-time studies leading to a bachelor’s degree eequalling180 ECTS (Öhlén *et al*., 2011; Råholm *et al*., 2010). As 50% of undergraduate nursing education consists of clinical practice, therefore, the relationship between the undergraduate program and nursing practice is considered to be of great importance (Maassen *et al*., 2011). The education program in Norway includes 50 weeks of clinical studies, 20 weeks in Community Health Care, and 30 weeks in various departments in hospitals (Ministry of Education and Reseach, 2017). In Norway, all registered nurses have an authorized responsibility with a mandatory commitment to supervise undergraduate nursing students in clinical practice (KD, 2001). Formal education as a supervisor or mentor is not compulsory for nurses in clinical practice. Research has found that supervisory skills and education are crucial to ensure the learning and allocation of knowledge and skills between supervisors and undergraduate nursing students (Severinsson, 2001; Sommer *et al*., 2020).

This study is a part of a larger project, ‘To learn there and stay there’, whose aim is to increase the quality of supervision to undergraduate nursing students at the Norwegian University of Science and Technology (NTNU). In Norway and other countries, there is a clear need for more nurses in primary health care (Romøren *et al*., 2011; Sogstad *et al*., 2020). The need for health professionals, especially nurses, will increase in both specialized and primary health care in the years to come (André *et al*., 2021; 2022). In Norway, the overall care policy both in specialized and primary health care is developed by the government, and overall resource allocation is largely controlled at the national level (Romøren *et al*., 2011). Still, the duty of organizing and delivering long-term care services is assigned to the local authorities in Norway’s municipalities (Sogstad *et al*., 2020). Primary health services are characterized by lower skills, where as much as 29% of the workforce is performed by workers without proper health professional education, mostly in long-term care (Romøren *et al*., 2011). When looking at salary and turnover for nurses in Norway, it seems like salary has little effect on turnover (Søbstad *et al*., 2021), and salaries in both specialized and primary health care in Norway are mostly similar.

The universities are also responsible for contributing to increasing the relation to start working in primary health care for undergraduate nursing students. The main aim of the project is to see if increasing competence in terms of education and sources available for the nurse supervisors will contribute to recruiting nurses to work in primary health care services.

In this study, we will focus on first-year undergraduate nursing students in their first clinical practice in nursing homes with the aim, to strengthen their supervision and learning. We have implemented an alternative model for supervision called, strengthened supervision during clinical practice in nursing education (SVIP). In Norway, both lecturers from the university and nurses from clinical practice supervise and assess the student in clinical practice (Zlamal *et al*., 2021; Aigeltinger *et al*., 2012; Warne *et al*., 2010). To prepare the nurses in clinical practice to supervise the undergraduate nursing students, three interventions are implemented; 1) a lecturer from the university organizes frequent seminars with the nurse supervisors 2) the nurse supervisors take part in three group counseling meetings with lecturers from the university, and 3) a reflection group for students and the lecturer from the university was formalized. The main elements were that the nurse supervisors were given enhanced accountability for assessing undergraduate nursing students’ performance in clinical practice.

For undergraduate nursing students to return to primary health care, the experience of the clinical placement in primary health care is rewarding, and getting better supervision must be one way to strengthen that (Warne *et al*., 2010; Leonardsen *et al*., 2021).

The aim of this study is:

To explore the association between the implementation of a new model of supervision and the impact of undergraduate nursing students‘ interest in working in primary health care.

## Method

A quantitative method was used to collect data. The questionnaire was developed based on the aims of the project and earlier existing studies.

### Setting

The setting for this study was the location of the undergraduate nursing student at NTNU. Around 500 undergraduate nurses are educated at NTNU each year. This study was aimed at undergraduate nursing students in their first year and students who had a clinical placement in nursing homes who together with the university participated in the implementation of SVIP. The undergraduate nurse education at NTNU is sited at three different campuses, Gjøvik, Trondheim, and Ålesund. The study aimed at implementing SVIP as a model for supervision for undergraduate nursing students. Personnel involved in this study included lectures, supervisors/nurses from clinical practice, researchers, and unit leaders.

### Sample

An invitation email was sent to 69 students who participated in the ongoing study for the implementation of SVIP in nursing homes. The invitation email included information about the purpose of the study and the topics for the survey (Polit and Beck 2012). All undergraduate nursing students who participated in the study during their clinical practice at NTNU were invited to participate in the study.

### Questionnaire

The questionnaire was developed for the study based on earlier research (Bogsti *et al*., 2013b; 2013a; Struksnes *et al*., 2012; Arvidsson *et al*., 2008; Nordhagen *et al*., 2013). The questionnaire consisted of 15 questions, 3 about the characteristics of the participants, 3 about the unit they were in practical placement, 3 about the relationship with contact lecturer, 3 about their relationship with the practical supervisor, two about the relationship with fellow students, and 1 about where they may consider starting work after graduation.

### Data collection

The lectures from the university responsible for the students at the participating nursing homes informed the student about the study and invited them to participate. The students received the link to the survey in their email.

### Data analyses

The data were collected in ‘Nettskjema’, the most secure and used survey tool in Norway (University of Oslo, 2020, accessed 27.20.2021). It is used for data collection in research and administrative tasks such as registrations, orders, and feedback from students and is a tool for the design and implementation of surveys, and online data collection for the university and college sector (University of Oslo, 2020, accessed 27.20.2021). The results were imported into SPSS (IBM, SPSS Statistics, 27). We used descriptive statistics to describe the respondents’ viewpoints on using SVIP as a model for supervision in clinical practice (Polit, 2012). For the correlation, we used Pearson’s correlation for a description of the relationship between the variables that describe what affects the students’ desire to work in primary health care, which is measured on the same interval or same ratio scale. It measures the strength of the relationship between the two continuous variables (Polit, 2012).

### Ethical considerations

Participation in the study was voluntary. All students received oral and written information about the purpose of the study and gave their consent to participate. The Norwegian Centre for Research Data approved the study on the 29 April 2021 (ref. no. 384451). To maintain the anonymity of each participant, the characteristic of the respondents such as age and gender are not used in further analyses.

## Result

Of 69 undergraduate nursing students who participated in the study, 31 answered the questionnaire, which gives a response rate of 45% (31/69). The undergraduate nursing student group consisted of 88% female and 13% male. The age range for this group revealed that 93% were from those aged 19–25 years and 6% whose ages ranged from 26 to 35 years. As many as 96 % follow the normal study progression, 13 % have completed a bachelor’s degree previously and just 3% study part-time. The undergraduate nursing students participating in this study are called respondents in the result and discussion part.

Table 1 shows that the respondents have a positive experience with the clinical practice site (42%) and the nurse supervisor in practice (52%). The impact of having a fellow student, or a peer, along with them in the primary health care setting was positively evaluated by the respondents (81%), while 42 % of the respondents rated that learning from peers was important. The responses related to the impact of the lecturer were more varied; however, 52% rated the experience as positive.

**Table 1.** Respondents’ response, in %, to extend of experience related to different questions, from 0 ‘negative experience’ to 5 ‘positive experience’.

Extend of experience/% respondents/question asked: To what extent did you experience -	0	1	2	3	4	5
The practice site as positive or negative?	0	0	3%	23%	32%	42%
Your relationship with the contact lecturer as positive or negative?	3%	7%	0	29%	35%	26%
Your relationship with the nurse supervisor as positive or negative?	0	13%	3%	10%	22%	52%
That your relationship with fellow students was positive or negative?	0	3%	0	0	16%	81%
That you and your fellow students learned from each other?	3%	3%	7%	16%	29%	42%

In Table 2, the respondents state the most important aspect of their perception of the clinical practice site as either positive or negative. Most of the respondents revealed the nurse supervisor as important for their perception of the clinical practice site (61%). The work environment was declared as important for 55 % of the respondents and the peers 42%. The lecturer has zero effect on the respondent’s perception of the clinical practice site.

**Table 2.** What was most important for your perception of the practice site as positive or negative? (The respondent might state three alternatives)

Alternatives	Present
The nurse supervisor	61.3 %
Work environment	54.8 %
Fellow students	41.9 %
Educational challenges	32.3 %
Closeness to the practice site	6.5 %
Contact lecturer	0 %

The respondents revealed that having good communication (84%) and professional safety (55%) were crucial for the development of a good relationship with the nurse supervisor. Regular meetings between the respondents and the nurse supervisor (3%) and spaciousness about attendance on the supervisor’s part (6%) were not declared as important for the respondents, as shown in Table 3.

**Table 3.** What was most crucial to your relationship with the nurse supervisor? (The respondent might state three alternatives)

Alternatives	Present
Good communication	83.9 %
Professional safety	54.8 %
Clear practical guidance	38.7 %
Roominess in relation to attendance	6.5 %
Regular meetings	3.2 %

In Table 4, the respondent rated the probability to work or undertake extra shifts at the clinical practice site. Only 23% stated that they would like to take an extra shift at the practice site, while 35 % rated some probability. When rating the probability after completing their undergraduate nursing program, whether they would want to work at the clinical practice site only 7 % rated high probability while 13 % rated some probability. As much as 26 % of the respondents rated that as a low probability of working in a primary health care setting.

**Table 4.** Respondents response, in %, to extend of probability related to different questions, from 0 ‘low probability’ to 5 ‘high probability’.

Extend of probability/% respondents/questions asked	0	1	2	3	4	5
To what extent is it likely that you want to take extra shifts at the practice site?	23%	3%	6%	10%	35%	23%
To what extent is it probable that after completing your education you want to work at the practice site?	26%	19%	6%	29%	13%	7%

The respondents should state where they plan or want to work after graduation. As much as 77 % stated that they plan to work in a hospital in specialized health care sector. Nursing homes and home care, which are in the primary health care sector, were only selected by 6% of the respondents each. Other options were also rated by the respondents; see Table 5.

**Table 5.** Regardless of how you have experienced the internship period, where have you envisioned that you will work after graduating? (The respondent might state three alternatives)

Alternatives	Present
Hospital, somatic	77.4 %
Rehabilitation center	19.4 %
Hospital, psychiatry	16.1 %
Home nursing	6.5 %
Nursing homes	6.5 %
District psychiatric center	3.2 %
Other	41.9 %

When assessing the significant correlation between the different variables, we found two significant correlations. The most interesting significant correlation is between ‘To what extent is it probable that after completing your education you want to work at the practice site?’ and ‘To what extent did you experience the practice site as positive or negative?’ with a significant correlation of ,475. We also found a significant correlation between ‘To what extent is it probable that after completing your education you want to work at the practice site?’ and ‘To what extend is it likely that you want to take extra shifts at the practice site?’ with a significant correlation of ,610. We also assessed if the relationship with fellow students would have any significant correlation to the other variables, but there were no significant correlations as shown in Table 6.

**Table 6.** Correlations (N=31)

Responses to questions To what extent-	Correlations	Is it likely that you want to take extra shifts at the practice site?	Is it probable that after completing your education you want to work at the practice site?	Did you experience that you and your fellow students learned from each other?	Did you experience he practice site as positive or negative?
Is it likely that you want to take extra shifts at the practice site?	Pearson’s correlation	1	,610^xx^	−,189	,341
	Sig. (two-tailed)		,000	,308	,060
Is it probable that after completing your education you want to work at the practice site?	Pearson’s correlation	,610 ^xx^	1	,095	,475^xx^
	Sig. (two-tailed)	,000		,611	,007
Did you experience that you and your fellow students learned from each other?	Pearson’s correlation	−,189	,095	1	,091
	Sig. (two-tailed)	,308	,611		,304
Did you experience the practice site as positive or negative?	Pearson’s correlation	,341	,475^xx^	,191	1
	Sig. (two-tailed)	,060	,007	,304	

xx Correlation is significant at the 0.01 level (two-tailed)

## Discussion

The purpose of this study was to explore the association between the implementation of a new model of supervision and the impact of undergraduate nursing students’ interest in working in primary health care. In the case of our study, the clinical setting was a nursing home.

### Perception of the clinical practice

Our findings showed that 74% of the respondents experience the practice site as positive or very good. In addition, our findings revealed that the nurse supervisor is perceived as the most crucial factor for the respondent’s perception of the practice site, but also the work environment plays a vital role. The work environment was also important for 55% of the respondent’s perception of the practice site. Other studies have pointed out that professional education aims to qualify candidates for professional practice, though the transition from education to work, and the work environment may have a significant role to achieve that (Warne *et al*., 2010; Begat *et al*., 2005). So, the work environment is important for the student’s learning conditions, and at the same time also essential for the student’s perception of the work site.

### Relationship with supervisor, fellow students, and teachers

Earlier studies have shown that adequate supervisory skills are vital to ensure learning, which is described as the transfer of knowledge and skills between supervisors and supervisees (Sommer *et al*., 2020; Severinsson, 2001). Effective communication (84%) and professional safety (55%) were most crucial for the respondents for the development of a good relationship with the practice supervisor in our study. However, clear practical guidance was also a key factor and may also be important in the transfer of knowledge between the nurse supervisor and the undergraduate nursing students. There is a need to support nurse supervisors to supervise and assess undergraduate nursing students in complex nursing practice (Leonardsen *et al*., 2021). The SVIP model has considered that and given more attention to supporting, educating, and tutoring the nurse supervisors, when offering three group counseling meetings with lecturers from the university. Our results seem to indicate that the SVIP model has succeeded in providing the nurse supervisors with more support and that the students are more satisfied with their clinical supervision. In our study, 74 % of the respondents reported that their relationship with the nurse supervisor was positive or very good.

Also, fellow students were important for the respondents, and 81 % experienced a positive relationship with their fellow students. The importance of fellow students for the perception of the practice site as positive was 41% so that it is an important factor for the well-being of the practice site for the respondents (Arkan *et al*., 2018). The peers a student has in clinical practice are essential for the learning process (Serçekuş and Başkale, 2016; Arkan *et al*., 2018), and 71% of the respondents in our study stated that they learned from fellow students. It seems like the implementation of the SVIP model has led to a closer relationship between the students. When students are placed together in clinical practice, it seems to be an important factor for the well-being at the practice site (Serçekuş and Başkale, 2016; Arkan *et al*., 2018), and it may lead to more recruitment for the primary health care sector.

The lecture’s influence on clinical practice was zero, which was a surprising finding. The relationship with the contact lecturer, on the other hand, was characterized by 61% of the respondents as very good or positive. This implies that the relationship with the contact lecturer is good, but the lecturer’s influence on the perception of the clinical practice site is insignificant, so the contact lecturer’s role in clinical practice must be discussed. The contact lecturer’s presence in clinical practice is not so important in the SVIP model. The most critical issue for the respondents to perceive the clinical practice site as positive or negative is the nurse supervisor (61%) followed by the work environment (55%). This is an important finding as we also found a significant correlation between how the student experienced the clinical practice site and to what extent they want to work at the clinical practice site after graduating. Studies have revealed that students may encounter problems in their relationship with the nurse supervisor (Warne *et al*., 2010; Noviani *et al*., 2022), while others have highlighted the importance of a positive learning environment for students to learn practical skills (Hayes *et al*., 2006; Arkan *et al*., 2018; Serçekuş and Başkale, 2016). In our study, we found that the nurse supervisor and the work environment are the most important aspects for the respondents to consider when they characterize the clinical practice site. Using the SVIP model, with the three group counseling meetings with lectures from the university, will therefore be important to strengthen the supervisor’s education and resources in their teaching of the students.

### Choosing to work in the primary health care setting

The results in this study revealed that 58% of respondents stated that it is high or some probability that they will take extra shifts at the clinical practice site in a primary health care setting. However, only 20 % stated that they have a high probability to start working at the practice site after graduation. Nursing homes and home care, which are in the primary care health care setting, were only selected by 6.5 % of respondents where they wanted to work after graduation. Since one of the main aims of our study was to explore if the implementation of a new model for supervision in clinical practice may encourage undergraduate nursing students to work in the primary health care sector, the finding was disappointing. Challenges for introducing changes in health care not only include the behavior and intentions of the health care personnel but also identifying motivational factors, such as focusing on the benefits of changes (André and Sjøvold, 2017; Schultz *et al*., 2017; Strobe, 2008). In our study, we have identified that the nurse supervisor was important for the respondent’s perception of the clinical practice site, and this influence may be used as a motivational factor for behavior changes among undergraduate nursing students. A positive attitude may influence an individual’s intention to change behavior, and the work environment can be used to determine readiness for change. So, it will be important in the future both to address attitude and work environment for undergraduate nursing students’ intention to work in primary health care following graduation. The role of the nursing supervisor and the work environment were the main findings in this study to influence on the respondent’s choice to work in primary health care following graduation.

### Limitations of the present study

This study has limitations. The study was conducted on a small group of students at three different campuses at NTNU. The implementation of SVIP may have been done differently on the different campuses. However, there was an effort to include frequent meetings to ensure that the model was used as similarly as possible to the other campuses. All data used in our study were based on self-reports, meaning that common method bias might be present (Podsakoff, 2003). Another limitation is the descriptive design, as it does not allow for causal explanations, and the questionnaire, which has not been validated. This must be taken into consideration when comparing the findings of this study with other studies. The sample was not powered; therefore, we cannot generalize the findings. Another limitation of the study relates to the setting. We have only used one setting (i.e., nursing homes) within the primary health care settings, so we cannot without reservations generalize to other settings.

## Conclusion

Our study has emphasized the importance of conducting follow-up research when changes are implemented to be able to assess the impact of the change using the SVIP model. In this study, the undergraduate nursing students responded about the implementation of a new model of supervision in clinical practice: the SVIP model. Our findings revealed that the work environment and the nurse supervisor are vital for the student’s learning and essential for the student’s perception of the work site in the primary health care setting. The results also appear to uncover that the SVIP model has succeeded in giving the nurse supervisors more support and tutoring, and the students are satisfied with the nurse supervisor.

Nevertheless, only a few students stated that they have a high probability to start working in the primary health care sector following graduation. Since one of the main aims of our study was to see if the implementation of a new model for supervising would encourage undergraduate nursing students to work in the primary health care sector following graduation, further research in this field is needed to explore factors that can facilitate undergraduate nursing students first designation choice following graduation to be primary health care sector.
